# Image-Based Computational Evaluation of the Effects of Atrial Wall Thickness and Fibrosis on Re-entrant Drivers for Atrial Fibrillation

**DOI:** 10.3389/fphys.2018.01352

**Published:** 2018-10-04

**Authors:** Aditi Roy, Marta Varela, Oleg Aslanidi

**Affiliations:** Department of Biomedical Engineering, School of Biomedical Engineering & Imaging Sciences, King’s College London, King’s Health Partners, St Thomas’ Hospital, London, United Kingdom

**Keywords:** atrial fibrillation, atrial wall thickness, fibrosis, modeling, MR imaging

## Abstract

**Introduction:** Catheter ablation (CA) is a common treatment for atrial fibrillation (AF), but the knowledge of optimal ablation sites, and hence clinical outcomes, are suboptimal. Increasing evidence suggest that ablation strategies based on patient-specific substrates information, such as distributions of fibrosis and atrial wall thickness (AWT), may be used to improve therapy. We hypothesized that competing influences of large AWT gradients and fibrotic patches on conductive properties of atrial tissue can determine locations of re-entrant drivers (RDs) sustaining AF.

**Methods:** Two sets of models were used: (1) a simple model of 3D atrial tissue slab with a step change in AWT and a synthetic fibrosis patch, and (2) 3D models based on patient-specific right atrial (RA) and left atrial (LA) geometries. The latter were obtained from four healthy volunteers and two AF patients, respectively, using magnetic resonance imaging (MRI). A synthetic fibrotic patch was added in the RA and fibrosis distributions in the LA were obtained from gadolinium-enhanced MRI of the same patients. In all models, 3D geometry was combined with the Fenton-Karma atrial cell model to simulate RDs.

**Results:** In the slab, RDs drifted toward, and then along the AWT step. However, with additional fibrosis, the RDs were localized in regions between the step and fibrosis. In the RA, RDs drifted toward and anchored to a large AWT gradient between the crista terminalis (CT) region and the surrounding atrial wall. Without such a gradient, RDs drifted toward the superior vena cava (SVC) or the tricuspid valve (TSV). With additional fibrosis, RDs initiated away from the CT anchored to the fibrotic patch, whereas RDs initiated close to the CT region remained localized between the two structures. In the LA, AWT was more uniform and RDs drifted toward the pulmonary veins (PVs). However, with additional fibrotic patches, RDs either anchored to them or multiplied.

**Conclusion:** In the RA, RD locations are determined by both fibrosis and AWT gradients at the CT region. In the LA, they are determined by fibrosis due to the absence of large AWT gradients. These results elucidate mechanisms behind the stabilization of RDs sustaining AF and can help guide ablation therapy.

## Introduction

Atrial fibrillation (AF), the most common sustained cardiac arrhythmia, is characterized by rapid and irregular activations of the upper chambers of the heart (Nattel et al., 2008). AF is independently associated with a twofold increase in all-cause mortality and increased morbidity, particularly stroke, heart failure, and cognitive impairment (Kirchhof et al., 2016). AF affects about 33.5 million people worldwide, and a progressive increase in the prevalence and incidence of AF, accompanied by high morbidity and mortality, is predicted over the coming decades (Chugh et al., 2014).

Catheter ablation (CA) is a well-established strategy for the restoration of sinus rhythm in AF patients who do not respond well to anti-arrhythmic drugs, with success rates of up to 70% in patients with episodes of AF lasting less than 1 week (paroxysmal AF). It typically relies on the insertion of a catheter into the atria, where it delivers high amounts of localized energy for the destruction and isolation of arrhythmogenic substrates without affecting the surrounding areas (Nattel, 2002). The widely accepted CA strategy to restore sinus rhythm is the electrical isolation of pulmonary vein (PV) sleeves in the left atrium (LA), which are believed to be the primary substrate for the generation of the ectopic beats and/or anchoring of re-entrant drivers (RDs) responsible for triggering and sustaining AF in the LA (Gray et al., 1998; Oral et al., 2002; Sahadevan et al., 2004). However, approximately 30% of AF patients are asymptomatic, which leads to delayed diagnosis and the development of persistent AF. In these patients, the success rate with CA drops to 42% (Ganesan et al., 2013), often requiring additional ablation procedures. A potential factor for the failure of CA in these patients is the presence of high degrees of electrophysiological and structural remodeling, which alters the AF substrate, making it harder to predict the locations of AF drivers. Therefore, by changing the focus of ablation strategies from anatomic to patient-specific functional targets, the efficiency of CA could be greatly improved.

Recent advances in catheter and electro-anatomic mapping technologies have enabled the development of more patient-specific ablation strategies that complement standard approaches, such as PV isolation (PVI), in persistent AF. These can directly target: (i) AF sources (ectopic triggers or RDs) identified invasively using basket catheters (Narayan et al., 2014) or non-invasively using body surface electrodes (Haissaguerre et al., 2014) and (ii) regions where the atrial substrate is expected to be arrhythmogenic, such as low voltage areas (Kottkamp et al., 2016). The role of RDs as drivers for AF has been long recognized (Gray et al., 1998; Sahadevan et al., 2004). However, RD-guided ablation is limited by the challenges of mapping and visualizing electrical activity on the endocardial surface with sufficiently high resolution. This can explain contradictory outcomes of multi-centre trials, some of which have shown favorable outcomes of RD-guided ablation (Miller et al., 2017), while others have failed to find advantages in this approach compared to PVI (Mohanty et al., 2018). However, with the advancement of novel imaging tools the paradigm is shifting toward non-invasive identification of patient-specific regions where the atrial substrate can be arrhythmogenic (Kottkamp et al., 2015; Cochet et al., 2018).

Atrial fibrosis is the most studied example of an arrhythmogenic substrate in AF patients and it has been reported to correlate with both AF incidence and post-ablation recurrence (McGann et al., 2014). Moreover, the level of fibrosis can be used for patient stratification, as CA procedures have a higher success rate in patients with less fibrotic burden (Mahnkopf et al., 2010). Recent clinical studies have reported that low-voltage areas on the endocardial surface represent abnormal atrial substrate caused by fibrosis and can be directly targeted by ablation (Blandino et al., 2017). While mapping RDs or low-voltage areas is invasive and time-consuming, fibrosis can be imaged noninvasively using late gadolinium enhancement (LGE) MRI (Siebermair et al., 2017). Modeling studies based on patient-specific LGE MRI reconstructions of fibrosis have suggested that slow conducting border zones around fibrosis (Morgan et al., 2016) are common anchoring sites for RDs. Moreover, Zahid et al. (2016) showed that RDs anchor at the border zone locations with specific spatial patterns of fibrosis. This suggests that the dynamics of RDs may depend on patient-specific fibrosis distribution.

In order to predict optimal target locations for ablation, a better understanding of the mechanistic influence of fibrosis on AF should be considered in combination with other structural factors, which have been shown to also influence the dynamics of RDs. Theoretical studies have highlighted the role of fiber orientation (Varela et al., 2016), surface curvature (Dierckx et al., 2013), and tissue thickness gradients (Biktasheva et al., 2015) on the dynamics of RDs. The prediction that RDs can drift and stabilize at borders between thin and thick tissue is arguably best supported by experimental evidence. Optical recordings in the sheep right atrium (RA) have shown that RDs tend to localize in bordering regions between thin and thick pectinate muscles (Yamazaki et al., 2012). Moreover, association of complex fractionated electrograms with atrial wall thickness (AWT) of the LA has also been reported (Park et al., 2014), pointing to the presence of AF substrate in these regions.

These findings show a potential of AWT gradients as a marker for identifying RD locations in the atria. However, neither the influence of AWT gradients on the RD dynamics in realistic atrial geometries nor comparative effects of AWT gradients and fibrotic patches on RDs have been investigated. Reconstruction of the atrial wall from imaging (Varela et al., 2017b) provides basis for computational modeling of the RD dynamics in patient-specific atrial geometries, to understand the role of AWT gradients in influencing RD locations. Elucidating the relationships between AWT gradients, fibrosis, and RD locations can lead to an improved understanding of AF mechanisms, and ultimately help identify patient-specific ablation targets, improving the efficacy of treatment.

This study aims to investigate the mechanistic influence of two structural factors: (i) AWT and (ii) fibrosis on the dynamics of RDs sustaining AF. To this end, computational simulations of atrial electrophysiology will be performed on (1) an idealized 3D atrial slab with a sharp change in thickness and (2) realistic RA and LA geometries obtained from MRI of six patients. Our working hypothesis is that competing influences of AWT gradients and fibrotic patches on conductive properties of atrial tissue determine anchoring locations of RDs in 3D atrial models.

## Materials and Methods

**FIGURE 1:**
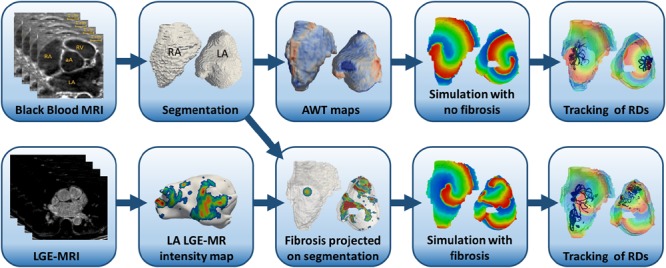
Workflow for the image-based atrial modeling (Studies 2 and 3). Top row: segmentation and registration of LA and RA geometries from black blood MRI used in simulations to investigate the role of AWT in the RD dynamics in the RA (Study 2a) and LA (Study 3a). Bottom row: patient-specific fibrosis distributions segmented from LGE MRI (bottom row) in the LA (Study 3b) and a synthetic fibrotic patch in the RA (Study 3a) used to evaluate the relative effects of fibrosis and AWT gradients on the RD dynamics in realistic atrial geometries.

The study consists of three parts summarized in **Table 1**. In Study 1, we perform simulations in an idealized 3D atrial slab with a varying thickness step to evaluate the mechanistic influence of AWT on the RDs dynamics (Study 1a). A single cylindrical fibrotic patch was subsequently included in the slab to investigate the competing effects of fibrosis and AWT (Study 1b). In Studies 2 and 3, we extend these simulations to realistic models of atrial geometries derived from MRI data, which were created using the general workflow shown in **Figure 1**. We first investigate the role of AWT and morphology of the RA (Study 2) and LA (Study 3) on the RD dynamics. The effect of a single cylindrical fibrotic patch on the RD dynamics in the RA is also investigated (Study 2b). Finally, we incorporate atrial fibrosis from patient-specific LGE MRI into our LA models to evaluate its influence on RDs relative to the effects of AWT and atrial geometry (Study 3b).

**Table 1:** Effects of AWT and fibrosis of the RD dynamics.

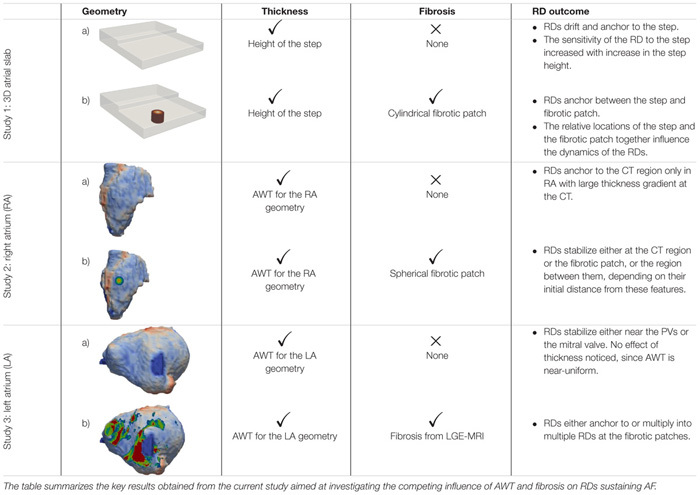

### Modeling Atrial Electrophysiology

All simulations were performed by solving the standard monodomain equation:

$$\begin{array}{c} \frac{\partial V}{\partial t}=\nabla \cdot (D\nabla V)-\frac{I_{\mathrm{ion}}}{C_{m}} \end{array}$$

Here, ∇ is the vector differential operator, *V* (mV) is the membrane potential and *t* is time (ms). *D* is the tensor with diffusion coefficients (mm^2^ ms^-1^) that characterizes the spread of voltage within the tissue, *C*_m_ (pF) is the membrane capacitance, and *I*_ion_ is the total membrane ionic current (pA). Our model was isotropic and the diffusion tensor was replaced by a scalar diffusion coefficient *D* = 0.1 mm^2^ ms^-1^, carefully chosen to match an atrial conduction velocity (CV) of 0.60 m s^-1^ typical of AF (Zheng et al., 2017). For *I*_ion_, we used the Fenton-Karma electrophysiology model (Fenton et al., 2008) modified to accurately described the restitution properties of remodeled atrial cells (Goodman et al., 2005). This atrial Fenton-Karma (aFK) cell model accurately captures the main characteristics of atrial action potentials and its restitution properties, while keeping the computational time relatively short. Equation (1) with no-flux boundary conditions was solved using forward Euler and centered finite differences schemes with temporal and spatial steps of 0.005 ms and 0.3 mm, respectively. In order to check independence of the simulation results on the choice of model, a subset of the simulations in Study 1 was repeated using the Coutemanche-Ramirez-Nattel (CRN) model (Courtemanche et al., 1998), which describes atrial myocyte in more detail. The latter has also been modified to match the restitution properties of remodeled atrial cells (Colman et al., 2013). The diffusion coefficient for the CRN model was chosen as 0.16 mm^2^ ms^-1^ to produce the atrial CV of 0.60 m s^-1^, same as that simulated with the FK model. The spatial step of 0.3 mm and temporal step of 0.005 ms were used for modified CRN model, for which numerical stability has been shown previously (Aslanidi et al., 2011).

Note that finite elements methods may have an advantage in terms of implementing zero-flux boundary conditions. However, benchmarks of cardiac electrophysiology models show that finite difference and finite elements solver have similar accuracy and convergence for spatial integration steps below 0.5 mm (Niederer et al., 2011). Moreover, both methods have been equally used for modeling of 3D atrial electrophysiology (Aslanidi et al., 2011; Zhao et al., 2017; vs. Roney et al., 2016; Zahid et al., 2016).

To model patchy fibrosis, we adopted the methodology from the study of Morgan et al. (2016). Each fibrotic patch was divided into five distinct levels representing increasingly severe fibrosis. The method for segmenting the fibrotic patches into these distinct levels for all the studies are described in their respective sections below. The diffusion coefficient *D* was progressively decreased by 16.67% to model the effect of fibrosis on slowing atrial conduction in fibrotic regions: level 0 corresponded to healthy tissue and *D* = 0.1 mm^2^ ms^-1^; inside the fibrosis patch, the value of *D* for levels 1-5 was 0.083, 0.067, 0.050, 0.033, and 0.017 mm^2^ ms^-1^, respectively. We did not used the value *D* = 0 for level 5, because there is no experimental evidence suggesting that dense fibrotic regions are completely non-conductive.

### Study 1: 3D Atrial Tissue Slab

Simulations were conducted on a 3D atrial slab of 200 × 200 × 25 voxels corresponding to tissue size of 60 mm × 60 mm × 7.5 mm with a surface area of 3600 mm^2^, similar to that of the LA (Varela et al., 2017a). High frequency sources of electrical activity have been observed in clinical studies in AF patients (Haïssaguerre et al., 1998; Sanders et al., 2005) with frequencies in a broad range from 5.5 to 10.5 Hz (corresponding cycle lengths, CL form 95-180 ms). The frequency of RDs in our simulations varied between 6 and 10 Hz, which agrees with the clinical observations. Given CV of 0.6 m/s, these CL values correspond to the wavelengths estimate (WL = CV × CL) between 57 and 108 mm. Therefore, the RD wavelength was comparable with linear dimensions of the slab and the LA.

**FIGURE 2:**
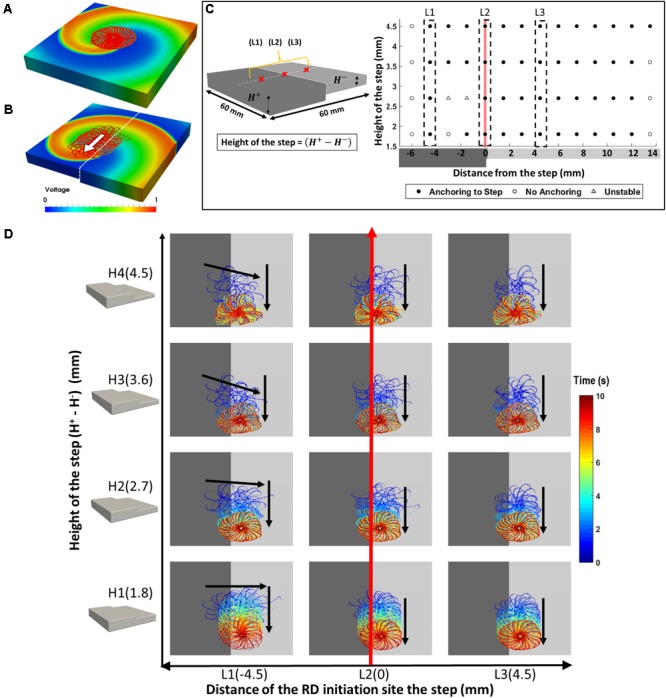
Effects of AWT step on the RD dynamics in atrial slab (Study 1a) with aFK model. Tip trajectories obtained by simulation of RDs at multiple locations on a 3D atrial slab with varying step height (H1-4): **(A)** RDs meander to form a flower-like pattern that was stationary in a slab with uniform thickness, but **(B)** in the slab with an AWT step, drifted toward and then along the step. **(C)** Outcome of the simulations for different RD initiation sites and step heights. **(D)** The RD tip trajectories for three initiation locations (L1-3): (L1) on the thicker side closer to the step – RD drifted toward the thinner side and then downward along the step, (L2) on the step – RD drifted down along the step, and (L3) on the thinner side – RD drifted toward the step and then down along the step.

**FIGURE 3:**
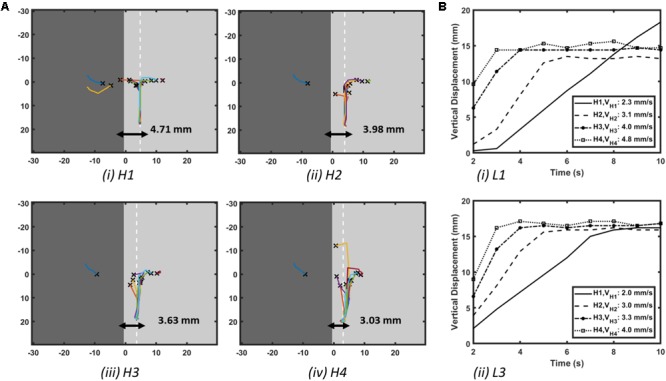
Effect of AWT gradients on the drift velocity of RD in atrial slab (Study 1a) with aFK model. **(A)** Trajectories of the core of RDs initiated at multiple locations on the slabs with varying step height (H1-4, see **Figure 2D**). Here, the X marks the site of the initial RD core for every location and the white dashed line in each slab indicates the location where the core of all the RDs stabilized after anchoring to the AWT step. **(B)** Vertical displacements of the RD core for locations L1 on the thick side and L3 on the thin side of the step (shown in **Figure 2D**) over 10 s are shown in panel **(Bi,ii)**, respectively, for each of the slabs with height H1-4. As the height of the step was increased, velocity of the vertical drift of the RDs also increased.

A thickness step was introduced at the middle of the slab, with thickness of the right-hand side fixed at 7.5 mm and that of the left-hand side was varied between 5.7 and 3 mm (**Figure 2**, H1-4). In Study 1a, we investigated the mechanistic influence of thickness gradients on the RD dynamics (**Figures 2, 3**). RDs were initiated using a cross-field protocol, where S2 pulse was applied at multiple distances from the AWT step, starting from 6 mm on the thick side to 13.5 mm on the thin side, and moving with a step of 1.5 mm (detailed information provided in the **Supplementary Material**). The trajectory of the RDs tip was tracked for 10 s in each simulation. Study 1a was also repeated using the CRN model to check the independence of results on the model choice (**Supplementary Figure S2**).

**FIGURE 4:**
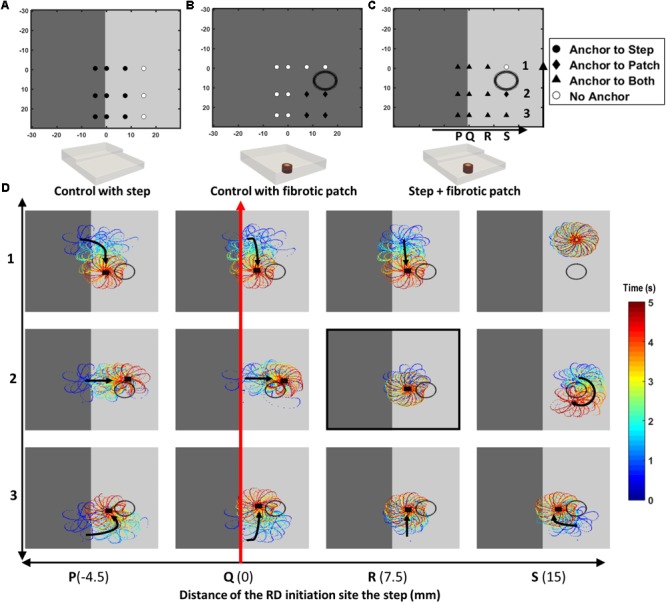
Competing effects of AWT and fibrosis in the 3D atrial slab (Study 1b). Outcomes of the RD simulations performed for 12 initiation locations (P1 to S3) with: **(A)** a step change in AWT, **(B)** a cylindrical fibrotic patch (shown with a black circle), and **(C)** both AWT step and fibrosis. In cases **(A,B)**, RDs anchored to the step (for locations marked with filled black circle) and fibrotic patch (for locations marked with black diamond), respectively. White circles mark locations where the RDs did not anchor to either. In case **(C)**, majority of the RDs anchored between the AWT step and fibrotic patch (for locations marked with black triangle). These RD tip trajectories on the top surface of the 3D slab are shown for the 12 locations (P1 to S3) in the panel **(D)**. Black arrows indicates the direction of RD core drift, and black squares marks locations where they became stationary and anchored.

In Study 1b, the comparative effect of AWT and fibrosis on the RD dynamics were also investigated by incorporating a cylindrical fibrotic patch of 9-mm diameter positioned 10.5 mm away from the AWT step on the thinner side (**Figure 4**). This was done for slab H2 with the step height of 2.7 mm. The rationale of placing the fibrotic region on the thinner side of the step was to mimic conditions when fibrotic tissue could be present in regions surrounding thick structures such as the crista terminalis (CT) in the RA. The cylindrical patch was divided into five concentric regions of gradually decreasing diameter representing increasingly dense fibrosis. RDs were initiated at multiple sites using the cross-field protocol, with varying relative distance from the step and the fibrotic patch: (i) 4.5 mm away from the step on the thick side (**Figure 4D**, column P), (ii) on the step (**Figure 4D**, column Q), (iii) 7.5 mm (**Figure 4D**, column R), and (iv) 15 mm (**Figure 4D**, column S) away from the step on the thin side. The RD tip trajectories were analyzed to explore their dynamics in each case. The choice of 4.5 mm as the extreme limit of the RD initiation distance to the left of the patch was to ensure that the RDs was sensitive only to the AWT gradient and not the fibrotic patch. Likewise, the choice of 15 mm as the extreme limit on the right side was to ensure the sensitivity of RDs only to the fibrotic patch.

### Studies 2 and 3: Right and Left Atria

Atrial geometries and fibrosis distributions were extracted from MRI to create 3D human atrial models, as described in previous studies (Varela et al., 2017b) and shown in the diagram in **Figure 1**.

#### Reconstruction of Atrial Geometries and AWT

Four healthy volunteers and two paroxysmal AF patients have been imaged (Varela et al., 2017b). Briefly, a black-blood MRI protocol (1.5 T, cardiac and respiratory gating, acquisition resolution: 1.4 mm × 1.4 mm × 1.4 mm) has been used to obtain patient-specific AWT maps non-invasively. Further details can be found in the previous publication (Varela et al., 2017b). The right (RA) and left atrial (LA) geometries of healthy volunteers and patients, respectively, were obtained by manual segmentation using ITK-SNAP (Kitware). A previously published method (Varela et al., 2017b) was used to generate patient-specific AWT maps, on which the computed RD tip trajectories were subsequently overlaid. In the RA, the region with the highest AWT (5.10 ± 1.00 mm against 1.8 ± 0.4 mm in the surrounding RA wall) can be identified as the CT (Sánchez-Quintana et al., 2002). We refer to this region as the CT in the following text. In some patients, this region was characterized by a large AWT gradient (4.75 ± 0.95 mm in the CT region against 2.91 ± 0.60 mm in the RA wall), whereas in other patients, the region was less prominent (2.90 ± 1.04 mm against 2.45 ± 0.54 mm in the RA wall).

#### Patient-Specific Fibrosis in the LA

The two paroxysmal AF patients have also undergone LGE MRI in the same scanning session and in the same phase of the cardiac cycle as the black-blood MRI acquisition, to detect fibrosis in the LA (1.5 T, cardiac and respiratory gating, acquisition resolution: 1.3 mm × 1.3 mm × 2 mm).

For Study 3b, the segmentation of fibrotic LA tissue was performed by analyzing the image intensity ratio (IIR), computed by dividing individual voxel intensities by the mean blood pool intensity. IIR values >1.08 were classified as interstitial fibrosis and >1.32 as dense fibrosis. The IIR threshold of 1.08 was obtained as an average of the previously proposed values of 1.2 (Benito et al., 2017) and 0.97 (Khurram et al., 2014). Transition from diffuse to dense fibrosis was represented by labelling segmented fibrotic tissue from 1 to 5 according to LGE-MRI intensity, where level 5 corresponded to dense fibrosis (regions with IIR > 1.32) and levels 1-4 corresponded to variable degree of diffuse fibrosis (regions with 1.08 < IIR < 1.32 split into four equal intervals). These fibrotic regions were then nonlinearly registered using MIRTK (Rueckert, 1999) and projected onto the LA geometries with transmural uniformity for the same patients using Paraview (Kitware) and Matlab (Mathworks, Inc.).

#### Synthetic Fibrosis in the RA

In the RA, due to lack of patient-specific fibrosis data, we added a synthetic 3D spherical fibrotic patch of diameter 9 mm, with similar labels 1-5 as done before for the 3D slab, in a region near the thick RA muscle bundle of the CT. This enabled a direct comparison of the effect of AWT and fibrosis on the RD dynamics in the RA (Study 2b). The choice of 9 mm diameter was based on calculating an average fibrotic patch size in the LA (Study 3).

#### Simulation Protocol for RD Initiation

A single RD was initiated using a cross-field protocol at different locations within the atrial geometries. In the RA, RDs were initiated in the vicinity of the CT region at six to nine different locations, where a large AWT gradient was typically observed. In the LA, four initiation locations were selected across the atrial wall. Simulations were performed in both geometries with and without fibrosis for a duration of at least 3 s. For each simulation, movement of the RD was tracked by recording location of its tip (organizing center around which a RD rotates) for each time step over duration of the entire simulation (Fenton et al., 2008). The resultant RD trajectories were then overlaid separately on the AWT and fibrosis maps for each patient-specific atrial geometry.

## Results

### Study 1: 3D Atrial Slab

The RDs initiated in the 3D atrial tissue slab meandered to form a characteristic hypotrochoidal (“flower-like”) pattern, with the RD tip moving along the “petals” in the outward clockwise direction around a central core. In a slab with uniform thickness, the flower pattern was stable, its radius was 10.4 mm, and the RD tip motion to complete the whole pattern took 500 ms (**Figure 2A**). However, with the introduction of a step change in AWT (**Figure 2B**), in addition to meandering around the core, the RD also drifted in a direction perpendicular to the direction of AWT change. The direction of RD drift also depended on its initiation location in the slab. The RD tip trajectories for multiple initiation locations are shown in the **Supplementary Figure S1** (H1-4), and the drift directions are summarized in **Figures 2C,D**.

The core of RDs initiated on the thicker side (L1) first drift toward the AWT step, eventually crossing it and drifting along it on the thinner side. The core of RDs initiated on the thinner side (L2-3), otherwise, drifted toward the step and along it, but did not cross over to the thicker side. Besides, RDs that were initiated far away from the step (**Supplementary Figure S1**) were not influenced by the AWT gradient and remained stationary. For the default aFK model settings, RDs anchored to the AWT gradient in the range from 1.8 to 4.5 mm (H1-4), when they were initiated less than 4.5 mm from the step on the thick side, or less than 12 mm (H1-3)/15 mm (H4) on the thin side. Moreover, after an RD anchored to the step, its core moved vertically along the step at a specific distance from it. The core always drifted on the thinner side, and its distance from the step decreased with increasing the step height (**Figure 3Ai–iv**).

Simulations of the 3D slab with the CRN atrial cell model demonstrated similar behavior of the RDs (**Supplementary Figure S2**). The RD tip in the CRN-based model also meandered to form a flower-like pattern with radius of 12 mm and one complete rotation around the pattern taking about 700 ms. Similar to the aFK-based model, anchoring of the RDs to the AWT step was observed, indicating that the anchoring phenomena was model independent.

The RD drift along the AWT step became faster as the height of the step (H1-4) was increased. The drift velocity (DV) was computed by measuring the vertical displacement of the RD core over time, which was done for each step height (H1-4) as shown in **Figure 3B**. DV was 2.3 mm/s for H1 and 4.8 mm/s for a larger step H4, both measured for the initial location L1 on the thick side. Similarly, for the initial location L3 on the thin side, DV was 2 mm/s for H1 and 4 mm/s for H4. Therefore, DV of the RD core increased with increasing the AWT gradient. Note that the direction in which the RD meanders to form the petals (clockwise in these simulations), and hence the direction of drift along the step (downward here) is determined by the cross-field initiation protocol. However, the anchoring of the RD to the thickness step does not depend on the wave’s chirality.

Results of the simulations comparing the effects of AWT and fibrosis in the 3D slab are shown in **Figure 4** for 12 different locations of the RD initiation, with the AWT step height (H2, 2.7 mm) and location of the synthetic fibrotic patch kept constant. In control cases of the AWT step only and the fibrotic patch only (**Figures 4A,B**, respectively), the RDs either drifted toward and anchored to the step (9 out of 12 locations) or anchored to the fibrotic patch and drifted in a clockwise direction around it (4 out of 12 locations). However, in the presence of both (**Figure 4C**), the majority of the RDs stabilized and anchored between the patch and the AWT step (8 out of 12 locations). Analyzing the RD trajectories in the latter case (**Figure 4D**), we found that RDs initiated on the thicker side, which were insensitive to the fibrotic patch alone, now drifted toward the patch located in the thinner region due to the influence of the AWT gradient. Once the RDs reached the region between the ATW step and the fibrotic patch, they could either drift downward along the step or drift rightward and rotate clockwise around the patch. Opposing influences from the AWT step and the fibrotic patch in this case can cancel each other out, with the RD becoming stationary and anchoring between the two structures.

For location R2 on the thinner side (highlighted in **Figure 4D**), increasing the step height from H2 to H4 caused the RDs to anchor to the step (**Supplementary Figure S3D**). However, increasing the distance between the step and the RD initiation site (from 7.5 to 15 mm), while preserving the distance between the initiation site and the patch, resulted in the RD anchoring to the patch (**Supplementary Figure S3E**).

### Study 2: Right Atrial Geometry

**FIGURE 5:**
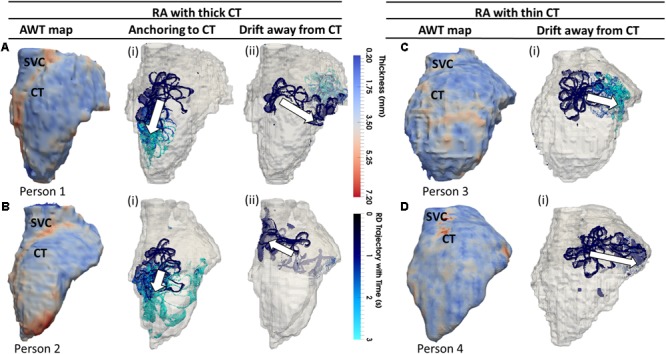
Effects of AWT on the RD dynamics on the RA. Trajectories of RDs (blue), initiated at four to nine locations in the RA, are overlaid on the AWT map for four different subjects. In Persons 1 **(A)** and 2 **(B)**, the CT region had a higher AWT compared to rest of the RA wall, while in Persons 3 **(C)** and 4 **(D)**, the CT region was not distinguishable by thickness. The majority of RDs initiated in Person 1 (six out of nine sites) and Person 2 (four out of nine sites) drifted toward the thick CT region and anchored to it **(Ai,Bi)**, but some also drifted toward the opposite side of the RA geometry **(Aii,Bii)**. However, none on four RDs initiated in Person 3 and Person 4 anchored to the CT region, drifting away toward the SVC or TCV instead **(Ci,Di)**. Here and below, white arrows indicate the direction of RD drift after initiation. More detailed illustration of RDs initiated at nine different locations in panel **(A)** is presented in **Supplementary Figure S4**.

The average AWT values computed in the CT region and the surrounding RA wall of Persons 1 (**Figure 5A**; CT: 3.45 ± 0.86 mm; RA: 2.09 ± 0.38 mm) and 2 (**Figure 5B**; CT: 4.75 ± 0.95 mm; RA: 2.91 ± 0.60 mm) showed a higher gradient in AWT compared to Persons 3 (**Figure 5C**; CT: 3.04 ± 0.65 mm; RA: 3.00 ± 0.54 mm) and 4 (**Figure 5D**; CT: 2.90 ± 1.04 mm; RA: 2.45 ± 0.54 mm). Hence, Persons 1 and 2 had a more prominent CT region compared to Persons 3 and 4.

In these patients, 55% of the RDs initiated at different locations drifted toward the CT region, either anchoring to it (**Figures 5Ai, 5Bi**) or crossing over it to the posterior side of the RA (**Figures 5Aii, 5Bii**). As an example, the 3D atrial voltage map in Person 1 is shown in **Supplementary Figure S3**. However, in the RA of Persons 3 and 4 (**Figures 5C,D**), the CT regions had thickness comparable to the remaining RA wall. Hence, the RDs neither drifted toward the CT, nor anchored at any location (**Figures 5Ci,Di**). Instead, they either drifted toward the superior vena cava (SVC) or the tricuspid valve (TSV) or terminated.

**FIGURE 6:**
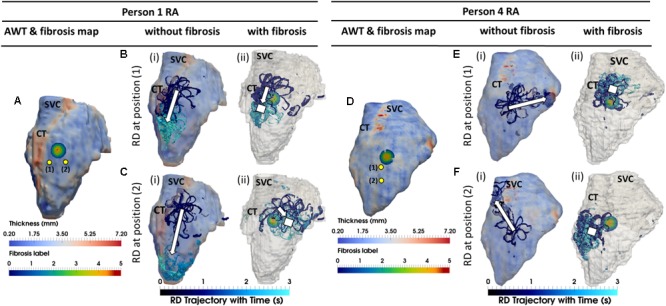
Competing effects of AWT and fibrosis in the RA. The RD trajectories (blue) in the RA, obtained by initiating RDs at two locations (yellow dots) without and with the presence of fibrosis are overlaid on the maps of AWT and fibrosis distribution, respectively, for Persons 1 **(A)** and 4 **(D)**. In Person 1 in the absence of fibrosis, RDs initiated at both locations **(Bi,Ci)** drifted toward the CT region with a large AWT gradient. However, in the presence of fibrosis, RD initiated at position 1 anchored between the CT and fibrotic patch **(Bii)**, and while RD initiated at position 2 anchored to the patch **(Cii)** which was located further away from the CT region. In Person 4 in the absence of fibrosis, RDs initiated at both positions **(Ei,Fi)** neither drifted toward the thin CT nor showed any anchoring. In the presence of fibrosis, RDs initiated at both positions **(Eii,Fii)** anchored to the fibrotic patch. SVC, superior vena cava. White squares mark locations where the RDs anchored.

**FIGURE 7:**
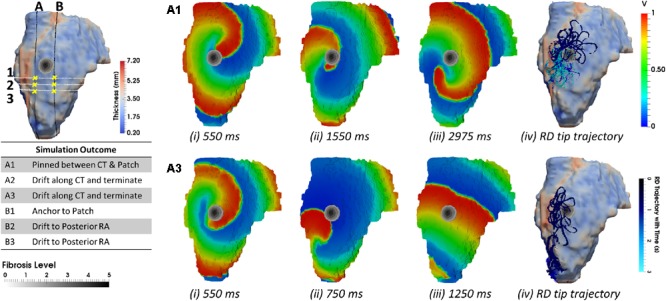
Anchoring of RD to AWT gradient at the CT region and the fibrotic patch in the RA of Person 1. Line A marks an approximate horizontal distance of 3 mm from the AWT gradient (CT region) and edge of the fibrotic patch, while Line B marks a horizontal distance of 13 mm from the CT region and 1 mm from the edge of the patch. The vertical distance of locations 1–3 from the center of the fibrotic patch was gradually increased (A1, 6.6 mm; A2, 10.2 mm; and A3, 13.8 mm), while distance from the CT region was approximately constant. The voltage maps for RD initiated at location A1 and A3 are shown in panels **(A1,A3)**, respectively, at different moments of time **(i–iii)**, and the RD tip trajectories are shown in **(iv)**. For the initiation location A1 close to the fibrotic patch, the RDs anchored between the CT region and the fibrotic patch **(A1)**. For the location A3 further away from the patch, the RDs drifted along the AWT gradient at the CT region toward the mitral valve **(A3)**. The outcomes of simulations for the RDs initiated at different locations are provide in the table.

When the RA simulations were repeated in the presence of a synthetic fibrotic patch, the RD trajectories were affected in all patients, as shown in **Figures 6, 7**. For the RAs with a prominent CT region (**Figure 6A**), RDs initiated closer to the fibrotic patch either stabilized between the CT region and the patch (**Figure 6Bii**), or anchored to the latter (**Figure 6Cii**), depending on the distance from their initiation site to either structure. The critical proximity to the CT region and the patch is challenging to determine in realistic RA geometry because, unlike AWT in a rectangular slab, the CT region is not a simple straight line. However, in an example provided for Person 1 (**Figure 7**), RDs initiated at the middle between edges of the CT region and the fibrotic patch (∼3 mm from either structure horizontally and 6.6 mm vertically) (**Figure 7**, location A1), stabilized and anchored between them. Increasing the horizontal distance between the RD and the CT to 13 mm (**Figure 7**, location B1) led to the RD anchoring to the fibrotic patch. However, in the RAs with no distinguishable thickness gradient at the CT region (**Figure 6D**), all RDs anchored to the fibrotic patch (**Figures 6Eii,Fii**).

### Study 3: Left Atrial Geometry

**FIGURE 8:**
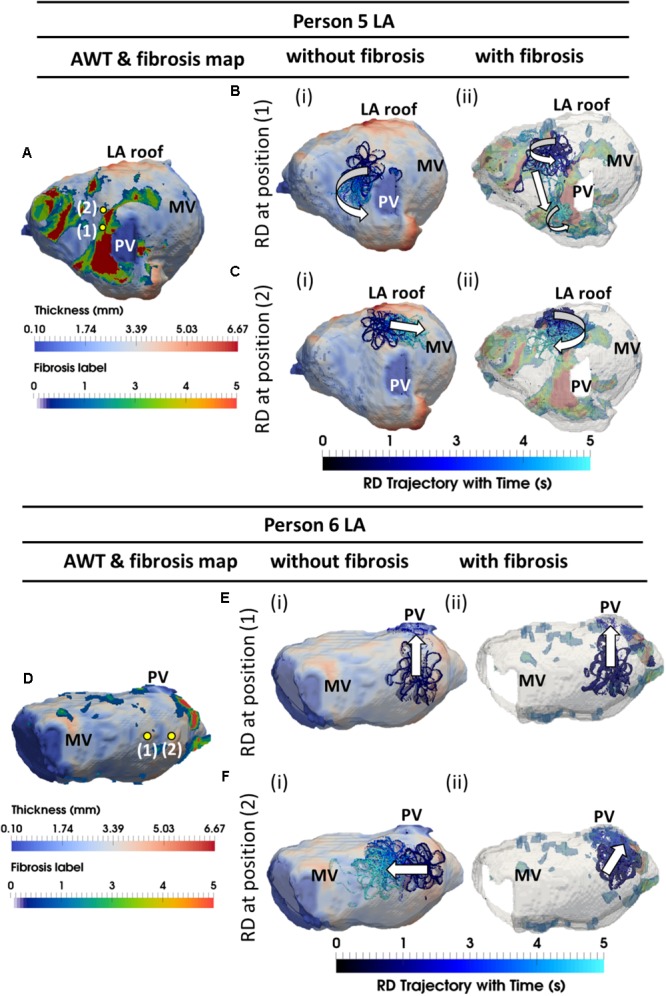
Competing effects of AWT and fibrosis in the LA. The RDs trajectories (blue) in the LA, obtained by initiating RDs at two locations (yellow dots), without and with the presence of fibrosis, are overlaid on the AWT map and fibrosis distribution **(A,D)**, respectively, for Person 5 (top) and 6 (bottom). In Person 5 in the absence of fibrosis, RDs initiated at positions 1 and 2 drifted toward the PVs [**(Bi** and LA roof **Ci)**], respectively. With additional fibrosis, RDs drifted from its initial location to a new location **(Bii)** for position 1 and anchored to the fibrotic patch **(Cii)** for position 2. In Person 6 without fibrosis, RDs initiated at positions 1 and 2 drifted toward the PVs **(Ei)** and MV **(Fi)**, respectively. With fibrosis, RDs continued to drift toward the PVs **(Eii)** for position 1, but anchored to the fibrotic patch **(Fii)** for position 2. PV, pulmonary vein; MV, mitral valve.

The AWT maps computed for LA geometry of Person 5 (**Figure 8A**) showed higher AWT in the LA roof (4.77 ± 0.60 mm) compared to the rest of the atrial wall (2.96 ± 0.31 mm). Therefore, in the respective simulations RDs drifted toward and along the LA roof (**Figure 8Ci**) when initiated in its proximity, but drifted toward the PVs (**Figure 8Bi**) if initiated further away. The LA of Person 6 (**Figure 8D**) was characterized by roughly uniform AWT (2.53 ± 1.20 mm), and RDs initiated in this geometry drifted either toward the PVs (**Figure 8Ei**) or the mitral valve (**Figure 8Fi**).

**FIGURE 9:**
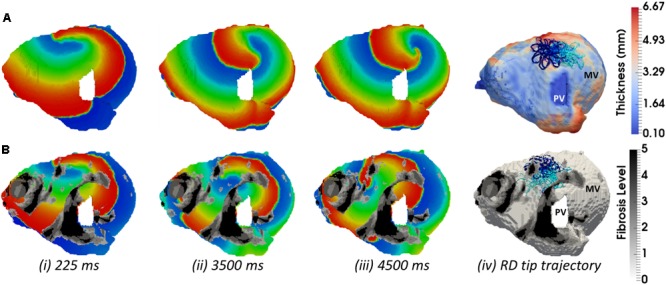
Competing effects of AWT and fibrosis in the LA. The voltage map for RD initiated at Position 2 (shown in **Figure 8A**) are shown at different moments of time **(i–iii)**. Rows **A** and **B** correspond to simulations without and with fibrosis, respectively. For each simulation, the RD tip trajectories (blue) are overlaid on their corresponding AWT and fibrosis map **(iv)**. **(A)** RD initiated without fibrosis drifts along the large AWT gradient at the LA roof, while in the presence of fibrosis **(B)**, the same RD anchors to the fibrotic patch.

In the LA, LGE MRI intensity-based segmentations yielded different fibrosis distributions in the two AF patients, as shown in **Figures 8A,D**. Both Persons 5 and 6 had fibrotic tissue in the LA regions surrounding the PVs, with a higher degree of fibrosis in one patient (14% of the atrial volume, **Figure 8A**) compared to the other (10%, **Figure 8D**). In Person 5, LA simulations repeated with the presence of fibrosis showed either stable RD anchoring to the border zones of fibrotic tissue (**Figure 8Cii**) or transient anchoring where RDs stabilized at a fibrotic patch in the first 1 s of simulation and then drifted to a new location in another fibrotic region and stabilized there (**Figure 8Bii**). 3D atrial voltage maps for the cases shown in **Figure 8Ci,ii** are illustrated in **Figures 9A,B**, respectively. In one particular case, we also observed the breakdown of the primary RD into multiple wavelets meandering between fibrotic patches (**Supplementary Figure S5B**). However, in Person 6, simulations repeated with the presence of fibrosis in the LA showed either stable RDs forming *macroscopic re-entry* around the PVs (**Figure 8Eii**) *or RDs* anchored to a fibrotic region (**Figure 8Fii**) near the PV.

## Discussion

The computational study performed on the 3D atrial tissue slabs and MRI-based realistic 3D atrial geometries enabled us to successfully evaluate the mechanistic influence of AWT and fibrosis distribution on the drift and anchoring of the RDs sustaining AF. In the 3D slabs, the AWT gradients acted as anchoring points for RDs in the absence of fibrosis, while additional fibrotic patches provided competing attractors for the RDs. The results obtained from the RA and LA model simulations confirmed that drift direction and anchoring points of RDs are determined by the interplay of competing influences of the patient-specific AWT gradients and fibrosis.

### Mechanistic Influence of AWT on the RD Dynamics

Simulations with both 3D atrial slabs and realistic 3D atrial geometries showed large AWT gradients provide anchoring locations for RDs. These results are in agreement with previous experimental observations in animal models of AF (Yamazaki et al., 2012). Moreover, larger AWT gradients have a stronger influence on the RD dynamics, forcing the RDs to drift with an increased velocity toward and then along the gradient. The underlying mechanism that enables the anchoring of RDs to these locations is likely to be based on a large source-to-sink mismatch in regions of high AWT gradient. The resulting current changes CV in tissue surrounding the AWT gradient, which helps stabilize the RDs in these locations. These results are in agreement with theoretical results using asymptotic theory based on simple three-variable ionic models (Biktasheva et al., 2015). We illustrated the tendency of RDs to anchor to regions with a high thickness gradient using the FK ionic model for remodeled atrial cells. Moreover, these results were reproduced using the CRN model that provides a detailed description of ionic currents and action potential in atrial cells (**Supplementary Figure S2**). This suggests that the anchoring of RDs to regions with large AWT gradients is model independent.

Note that, in addition to the influence of AWT gradients, anchoring of RDs to junction between thin and thick tissue observed by Yamazaki et al. could also be explained by the presence of pectinate muscles and activity of stretch-activated channels. Recent evidence also suggest that AF substrate can be provided by 3D structure of the atrial wall, which may cause epicardial and the endocardial discordance (Gutbrod et al., 2015). The latter may be explained by the formation of transmural RDs stabilized by interstitial fibrosis in the network of pectinate muscles (Hansen et al., 2015).

In this study, image-based RA and LA geometries from four healthy volunteers and two paroxysmal AF patients were used to assess the link between RD activity and regions of large AWT gradients. The subject-specific AWT was computed using previously published protocol from Varela et al. (2017b). Our results showed that RDs anchored to the CT region in subjects with a large AWT relative to the surrounding RA tissue. This findings can explain the presence of RD activity at the CT region, which has been reported in previous clinical (Kalman et al., 1998) and modeling (Gonzales et al., 2014) studies. However, RDs in this region can also be generated due to its high anisotropy (Aslanidi et al., 2011). In the atria with more homogeneous AWT (two RA models and all LA models), there was no clear link between the RD dynamics and specific AWT features. Instead, the direction of RD drift was always toward the TSV in the RA or the PVs and MV annulus in the LA. Our findings suggest that information from AWT may aid in designing personalized ablation strategies in patients whose atria are characterized by large gradients in thickness. Patient MRI can be used to reconstruct 3D atrial structure and identify patient-specific areas of large AWT gradients and fibrosis prior to CA procedures. Patient-specific models built based on such data can then predict the likely RD locations, and therefore aid in selecting the optimal ablation strategy for a given patient.

### Competing Influence of Fibrosis and AWT on RDs

Our 3D model simulations using both atrial slabs and realistic atrial geometries revealed the interplay between the effects AWT and fibrosis on the dynamics of RDs. Previous imaging-based computational studies have reported anchoring of RDs at fibrotic patches in the LA (Gonzales et al., 2014; Morgan et al., 2016; Zahid et al., 2016). However, to the best of our knowledge, our study is the first to explore the competing effects fibrosis and AWT gradients on the dynamics of RDs in both atria.

Simulations performed in both the 3D slab and realistic RA geometries produced similar results, with RDs anchoring between a region with a large AWT gradient (such as the CT region) and local fibrosis patches. Hence, locations of the RDs stabilization can be strongly influenced by both the presence of fibrosis, as predicted by previous studies (Morgan et al., 2016; Zahid et al., 2016), and the AWT gradients in the atria. These results can be explained by relative effects of (i) fibrotic patches providing slow conduction zones anchoring RDs and (ii) large AWT gradients creating substantial source-to-sink mismatches. The latter facilitate faster condition in the direction along the gradients. As the drift velocity of RDs along the AWT gradients increases with the increased gradient magnitude (**Figure 3B**), the RDs are more likely to drift along a large gradient, anchoring to it. Therefore, atrial tissue regions between a fibrotic patch and a large AWT gradient experience both competing influences and are most likely to anchor RDs. In addition, our simulations in both 3D slab and RA showed that RDs anchored to the fibrotic patch only when they were initiated further away from the structural features (thickness step, CT region) with large AWT gradients. Hence, anchoring of the RDs to either the AWT gradients or fibrotic patches may depend on (1) their comparative ability to provide suitable substrate and (2) their relative distance from the RD initiation site.

Our results could explain why the correlation between LGE-derived fibrosis and RDs locations reported by Cochet et al. (2018) was weaker in the RA than the LA. This may be explained by the higher variability in AWT in the RA compared to the LA, with the AWT gradients in the RA providing alternative anchoring locations for the RDs. Note that in this study, we used a synthetic cylindrical fibrotic patch in the RA, which was a simplified approximation of fibrotic patches of irregular geometry obtained from patient LGE MRI. The lack of patient-specific RA fibrosis data in our study was due routine pre-ablation LGE MRI scans being restricted to the LA. Previous studies (Zahid et al., 2016) have linked RD behavior to the heterogeneity of the shape of the fibrotic patch. While a simple cylindrical patch in the RA was not patient-specific, using it enabled us to focus on the relative mechanistic influences of slow-conducting fibrotic tissue and AWT gradients, without considering additional complex effects of the fibrosis shape.

In LA model simulations in the absence of patient-specific fibrosis distribution, RDs were mostly localized in regions surround the PVs and MV openings. Such a lack of influence of AWT on the RD stabilization in the LA could be explained by the absence of structural features with large AWT gradient in most patients. The only exception was Person 5, where the RD anchored to a large AWT gradient at the LA roof. However, when the LA simulations were repeated with LGE MRI-derived fibrotic patches, a clear stabilization of RDs near the patches was observed in all patients. In the exceptional case of Person 5 (**Figure 8**), who had high fibrosis burden, RDs were unstable and formed multiple wavelets meandering between fibrotic patches.

These results contribute to the understanding of why PVI is effective for paroxysmal AF patients with low fibrosis burden (Akoum et al., 2011). Even in paroxysmal AF, the number of RDs near the PVs is higher than the number of focal triggers (Narayan et al., 2013) – the presence of RDs near the PVs, and therefore the success of PVI, in those patients may be explained by the lack of other anchoring locations, such as large AWT gradients or fibrotic patches. However, for AF patients with high fibrosis burden, the altered atrial substrates can result in the drift and multiplication of RDs in regions of slow conduction, such as the border zones of fibrotic tissue (Morgan et al., 2016). Therefore, assessment of patient-specific fibrosis distribution in the LA of persistent AF patients, facilitated by LGE MRI, may assist in the prediction of RD locations. In future, this could be tested by a retrospective study on a patient cohort that have been successfully ablated after LGE MRI mapping of fibrosis.

### Limitations

In our study, fibrosis was modeled as regions of progressively slow conduction linked with LGE MRI intensity. The LGE MRI threshold of 1.08 IIR (level 1) and 1.32 IIR (level 5) was obtained from previous studies validated by electro-anatomical mapping data (Khurram et al., 2014; Benito et al., 2017), but the IIR threshold values for splitting the border zone into levels 1-4 was not validated due to lack of experimental data. However, correlation between decrease in CV and increase in IIR has been reported (Fukumoto et al., 2016). Therefore, our approach of gradually decreasing CV across fibrosis levels 0-5 is in agreement with patient studies. Recent studies have also shown that additional small effects of fibroblast-myocyte coupling (Morgan et al., 2016) and paracrine effects (Roney et al., 2016) could also influence the RD stabilization. However, data on such effects in human are unavailable, and modification of the model parameters based on *ex vivo* animal data can produce results that are inconsistent with clinical data (Roney et al., 2016). Therefore, such effects were not considered in this study.

Our study did not consider the influence of other patient-specific factors such as atrial anisotropy and electrophysiological heterogeneity, which may contribute to drift of the RDs observed in the realistic LA and RA geometries (Varela et al., 2016). Atrial fiber orientation is known to be complex (Ho and Sánchez-Quintana, 2009) and can have significant effects on atrial conduction (Aslanidi et al., 2011; Varela et al., 2016). However, fiber orientation was not incorporated in this study due to the absence of patience-specific data. Future studies will aim to incorporate information about fiber orientation into patient-specific atrial models based on recently proposed rule-based approaches (Fastl et al., 2018).

Note also that aFK model developed by Goodman et al. was based on data from tachypaced sheep atrial cells, due to a lack of equivalent human data. However, comparison of APD between the aFK model and in the CRN model for a remodeled human atrial myocyte showed a good agreement between the two models: less than 5% difference in APD in the considered range of frequencies between 6 and 10 Hz. Moreover, the main result of the study – that RDs anchor to regions with large AWT gradients – was qualitatively similar between the aFK and CRN models.

Finally, LA wall dilation and heterogeneous wall thinning can occur with the disease progression from PAF to permanent AF (Nakamura et al., 2011). Therefore, results of this study, which are based on data from healthy volunteers and PAF patients, cannot be extended to persistent AF. Future studies will aim to characterize AWT changes in persistent AF and evaluate the arising effects on AF drivers.

## Conclusion

This study elucidated the role of AWT as a substrate for RDs and marker for the identification of RD locations in the patient-specific atria, and compared the AWT effects with the respective effects of fibrosis. In the RA, RDs stabilized around structural features with large AWT gradients, while the addition of fibrotic patches provided an alternative attractor for the RDs. In the LA that had more uniform AWT distributions, RD locations were determined by the distribution of fibrotic patches or by anatomical features (e.g., the PVs). These findings corroborate our hypothesis that anchoring locations of RDs are dependent on the relative influence of gradients in AWT and fibrosis, and suggests the non-invasive assessment of AWT and fibrosis using MRI may inform clinical interventions in AF patients.

## Author Contributions

All authors have made substantial contributions to this study. AR and OA conceived and designed the study, and drafted the manuscript. AR substantially contributed to data analysis and computer simulations, and OA substantially contributed to the interpretation of the results. AR, MV, and OA contributed to data analysis, computer simulations, and manuscripts editing. All authors have also approved the final version to be published while agreeing to be accountable for all aspects of the work in ensuring that questions related to the accuracy or integrity of any part of the work are appropriately investigated and resolved.

## Conflict of Interest Statement

The authors declare that the research was conducted in the absence of any commercial or financial relationships that could be construed as a potential conflict of interest. The reviewer PB and handling Editor declared their shared affiliation.
