# Supplementary material for: Image-Based Computational Evaluation of the Effects of Atrial Wall Thickness and Fibrosis on Re-entrant Drivers for Atrial Fibrillation
Source: Front Physiol. 2018 Oct 4;9:1352. doi: 10.3389/fphys.2018.01352 (PMC6187302; doi:10.3389/fphys.2018.01352)
Supplement: Supplementary file 1 [file Data_Sheet_1.docx]

Supplementary Material

Image-Based Computational Evaluation of the Effects of Atrial Wall Thickness and Fibrosis on Re-Entrant Drivers for Atrial Fibrillation

Aditi Roy, Marta Varela, Oleg Aslanidi

Department of Biomedical Engineering, School of Biomedical Engineering & Imaging Sciences, King’s College London, King’s Health Partners, St Thomas’ Hospital, London, SE1 7EH, United Kingdom.

*** Correspondence: Oleg Aslanidi**School of Biomedical Engineering & Imaging Sciences
King’s College London
London SE1 7EH, United Kingdom
oleg.aslanidi@kcl.ac.uk; Phone: +44(0)2071887188

# Stimulation protocol for Study 1 (3D Slab)

RDs were initiated using the cross-field protocol. S1 stimulus was applied by introducing a voltage step (voltage: 1 (aFK) and 20 mV (CRN); duration: 1 ms) to part of the tissue slab 0.15 x 60 x 7.5 mm^3^, initiating a plane wave with the wavefront parallel to the AWT step. After 70ms, S2 stimulus was applied in the direction perpendicular to the wavefront, by resetting the voltage (voltage: 0 (aFK) and 20 mV (CRN); duration: 2ms) in part of the tissue 60 x (30 + X) x 7.5 mm^3^. The duration of 70 ms and the specific stimuli locations were chosen to keep RDs away from the boundary of the 3D tissue slab, to focus on its interaction with the AWT step. In Study 1a, the location X was varied from 7.5 mm to 0 mm in steps of 1.5 mm on both sides of the step. In Study 1b, the location X was -4.5 mm, 0 mm, 7.5 mm and 15 mm (Supplementary Figure 1).

# Stimulation protocol for Study 2 (RA) and 3 (LA)

RDs in the RA were also initiated using the cross-field protocol. Here, S1 stimulus (voltage: 1; duration: 1ms) was applied along a plane parallel to the superior vena cava, and after 50 ms S2 stimulus (voltage: 0; duration: 2 ms) was applied in a plane perpendicular to that of S1. For each of the 4 RAs, the locations of S1 and S2 were varied such that the intersection between the two planes was located near the CT region. An example for Person 1 in Study 2(a) is shown in Figure 4, where 9 such locations have been marked with yellow crosses. Likewise, in Study 2(b), initiation locations were chosen such that in some cases the distance from the AWT gradient is fixed but the distance from the fibrotic patch is varied, and in others vice versa. An example for Person 1 is shown in Figure 5. In Study 3 for the LA, RDs were initiated with similar S1-S2 protocol as in the RA with and without fibrosis, at different locations selected throughout the atrial wall. An example for Person 5 is shown in Figure 6.

## Supplementary Figures

**
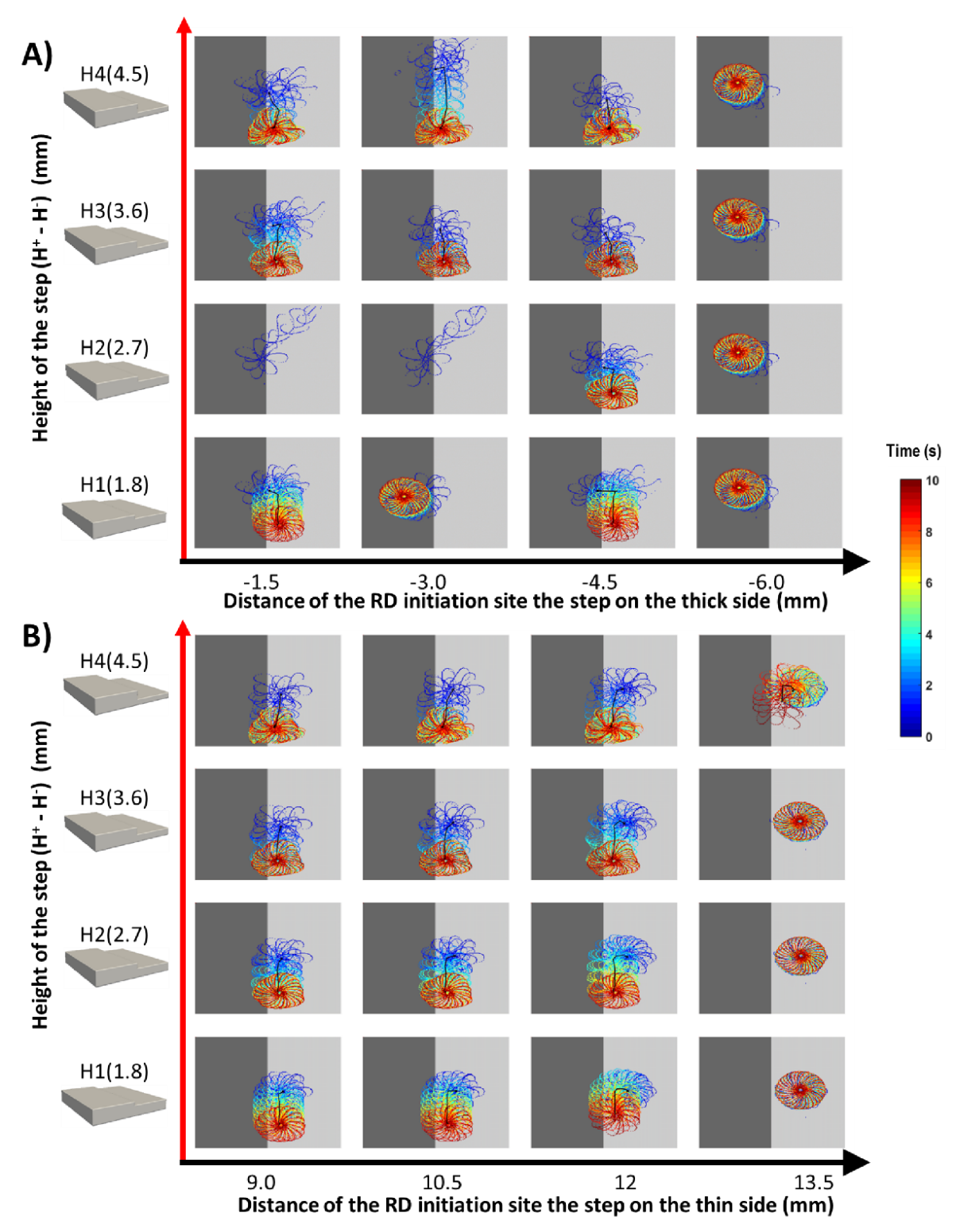
**

**Supplementary Figure 1.** Effects of changes in AWT step height and RD initiation location on the anchoring of RDs in 3D atrial slab (Study 1a) with aFK model. Tip trajectories obtained by initiating RDs at multiple locations on the thick (A) and thin (B) side of the 3D slab, while varying the height of the step (H1-4). (A) RDs initiated on the thick side of the slab first drift towards the AWT step, eventually crossing it and drifting along it on the thinner side with exception to H2, where the RDs terminated. (B) RDs initiated on the thin side drifted towards the step and along it, but didn’t cross over to the thicker side. Note that the core of the RDs initiated beyond 4.5 mm on the thick side and 12 mm (H1-3) / 15 mm (H4) on the thin side were not sensitive to the step (panels on the right).


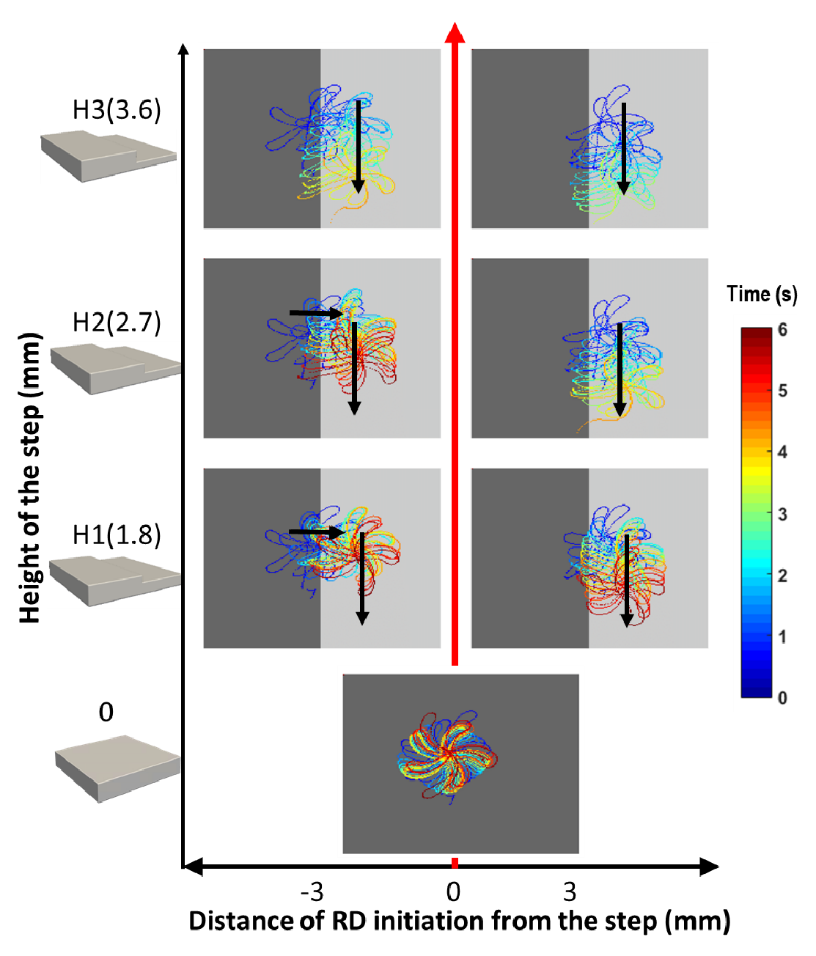


**Supplementary Figure 2.** Effects of AWT step on the RD dynamics in 3D slab with the CRN atrial cell model (Study 1a). The characteristic flower-like pattern formed by the RD tip was stationary on a uniform thickness slab (bottom panel) and drifted towards and then along the step for the slab with AWT gradient (other panels). The core of RDs initiated on the thicker side of the 3D slab crossed over to the thinner region before drifting along the step. Black arrows indicate the direction of the drift.


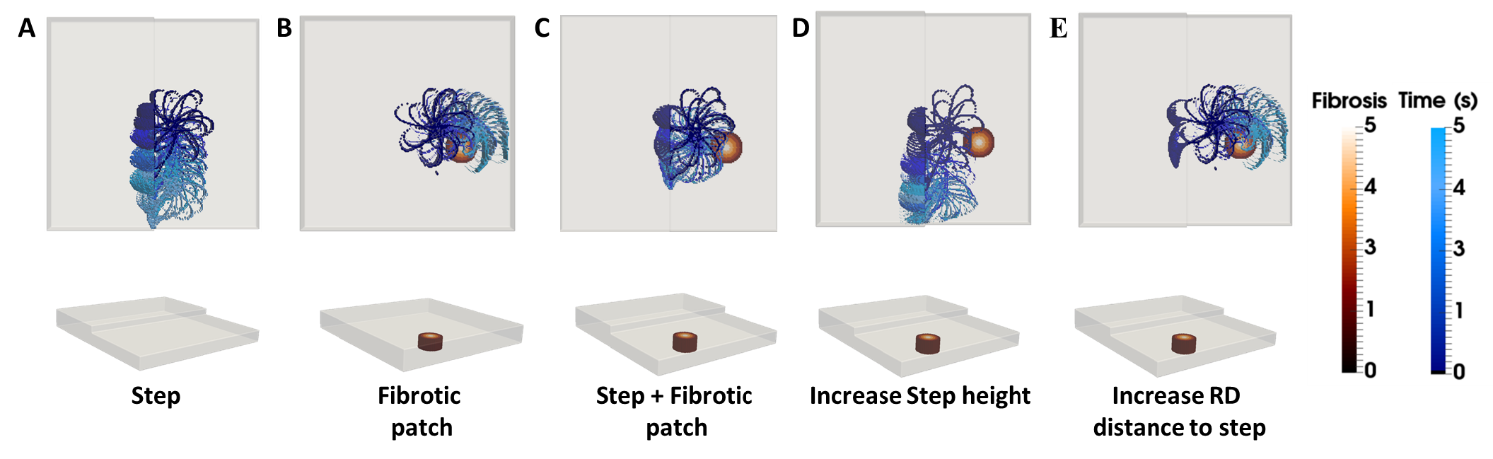


**Supplementary Figure 3.** RD tip trajectories with the AWT step and fibrosis in a 3D atrial slab. The trajectories are shown for the initiation location R2 (Figure 4D of the main manuscript) in the slab with AWT step only (A), fibrotic patch only (B), and both (C). In the first two cases, the RD anchored to the step and the fibrotic patch, respectively. However, in the latter case the RD stopped drifting and stabilized between the step and the fibrotic patch. Increasing the step height from H2 to H4 resulted in the RD drifting to the step (D), while increasing the distance from the step to the RD initiation site (from 7.5 mm to 15 mm) resulted in the RD drifting to the fibrotic patch (E).


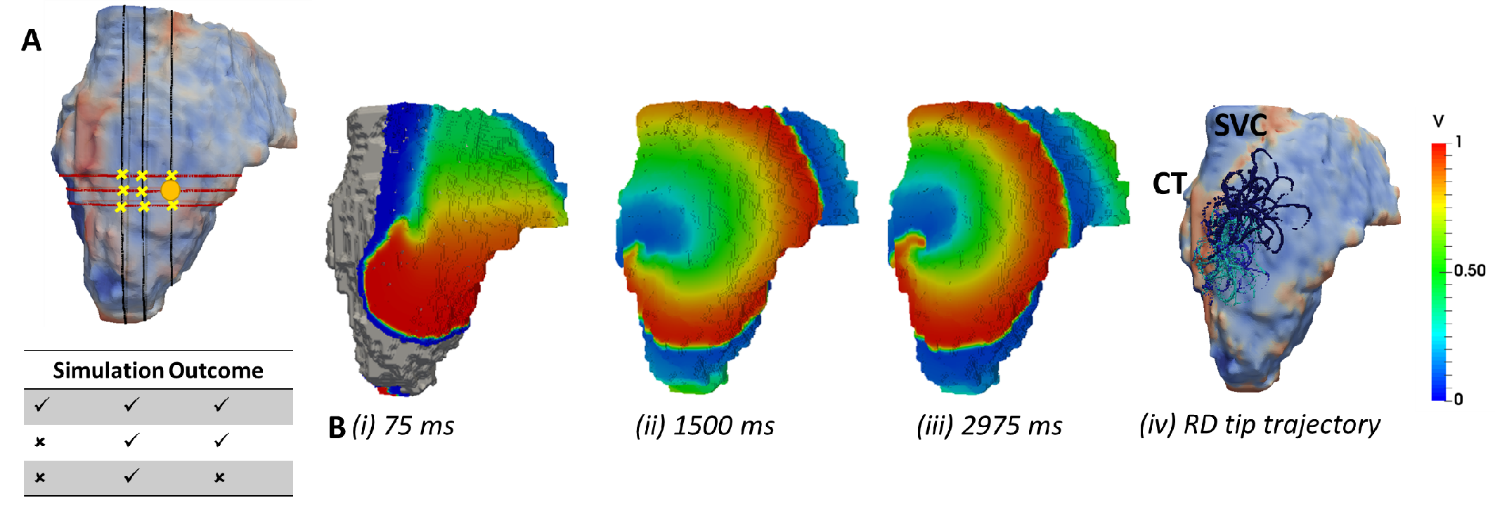


**Supplementary Figure 4.** Anchoring of RD to AWT gradient at the CT region in the RA of Person 1. A) All RD initiation locations are marked by yellow crosses, and in the table below ✓ corresponds to anchoring to the CT region and 🗶 to no anchoring. The voltage maps for RD initiated at the location marked by yellow circle is shown in panel B (i-iii) for different moments of time, and the complete tip trajectory with the RD anchored to the CT region is shown in panel B (iv).


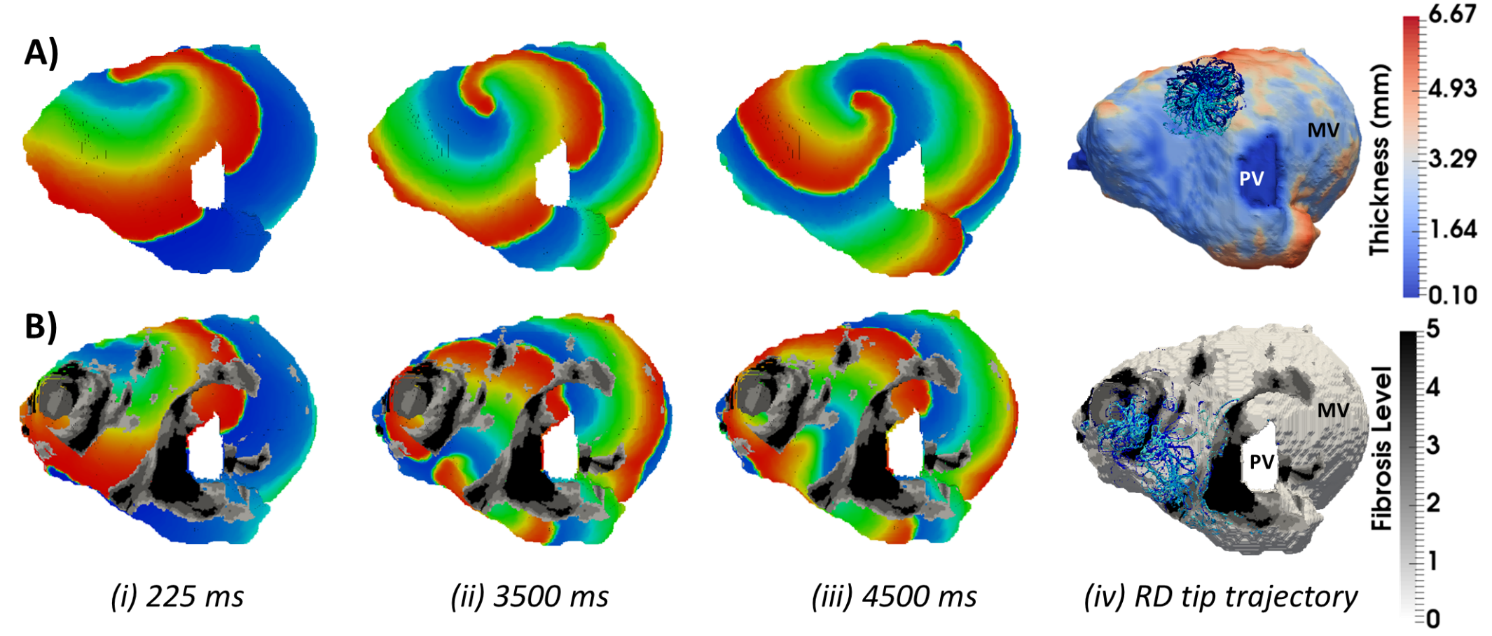


**Supplementary Figure 5.** Competing effects of AWT and fibrosis in the LA. The voltage maps for RDs are shown at different moments of time. Row A correspond to simulations without fibrosis and row B correspond to simulations with fibrosis. For each simulation, the RD tip trajectories (blue) are overlaid on their corresponding AWT and fibrosis map (v). RD initiated without fibrosis (A) anchors between the PV and a large AWT gradient at the LA roof, while in the presence of fibrosis (B), the same RD becomes unstable and breaks into multiple wavelets meandering between fibrotic patches.
